# 

**Published:** 1976

**Authors:** 


Bristol Medico-Chirurgical Journal Vol. 91 No. 339/340

### Contents

| | |
|---|---|
| Obituary — R. C. Wofinden ... ... | 3 |
| Low Dose Insulin Regimen for Diabetic Coma ... ... ...<br>Dr. C. M. Asplin | 5 |
| The Clinical Development of Disopyramide ... ... ... ...<br>Dr. S. I. Ankier | 11 |
| The Role of Disopyramide after Myocardial Infarction ... ...<br>Dr. M. B. S. Jones | 12 |
| University of Bristol Examination Results | 13 |

Printed by F. Bailey & Son Ltd., Dursley, Glos.

